# Supplementary material for: Nurses’ perspectives on the provision of self-care management education to patients with heart failure: A qualitative study at Cardiac Hospital, Dar es Salaam Tanzania
Source: PLoS One. 2024 Jul 12;19(7):e0306459. doi: 10.1371/journal.pone.0306459 (PMC11244802; doi:10.1371/journal.pone.0306459)
Supplement: S1 Table — (DOCX) [file pone.0306459.s001.docx]

**PONE-D-23-35648R1**

Nurses’ perspectives on provision of self-care management education to patients with heart failure. A qualitative study at Cardiac hospital, Dar es Salaam Tanzania

According to PLOS one here is the data set for the article titled above.

A total of twelve (12) Registered Nurses participated in the study.

| Participants ID | Age (Years) | Sex | Education Level | Experience (Years) |
| --- | --- | --- | --- | --- |
| P1 | 40 | Male | Bachelor Degree | 6 |
| P2 | 36 | Male | Bachelor Degree | 6 |
| P3 | 32 | Male | Bachelor Degree | 4 |
| P4 | 33 | Male | Bachelor Degree | 4 |
| P5 | 31 | Male | Bachelor Degree | 4 |
| P6 | 33 | Female | Diploma | 7 |
| P7 | 32 | Female | Diploma | 7 |
| P8 | 34 | Female | Diploma | 7 |
| P9 | 33 | Female | Diploma | 4 |
| P10 | 35 | Female | Diploma | 7 |
| P11 | 32 | Female | Diploma | 4 |
| P12 | 36 | Female | Bachelor Degree | 4 |

**Investigator**

Peter M. Shirima

Advanced Nurse Practitioner MNH-Mloganzila
