# Supplementary material for: Nurses’ perspectives on the provision of self-care management education to patients with heart failure: A qualitative study at Cardiac Hospital, Dar es Salaam Tanzania
Source: PLoS One. 2024 Jul 12;19(7):e0306459. doi: 10.1371/journal.pone.0306459 (PMC11244802; doi:10.1371/journal.pone.0306459)
Supplement: Attachment — Submitted filename: Response to Reviewers .docx [file pone.0306459.s002.docx]

Peter M.Shirima

P.O.Box 65000

Dar es Salaam, Tanzania

Email;pkasongoro@gmail.com

Chief Editor,

PLOS ONE Journal,

Section of Cardiovascular Medicine and Nursing.

**Dear Editor**,

RE: **RESPONSE TO REVIEWERS ARTICLE NO. PONE-D-23-35648**

We wish to re-submit an original qualitative research article entitled “Nurses' perspective on Provision of self-care management Education to patients with heart failure” for consideration by PLOS One Journal.

1. As I stated in the previous submission, this research receives limited fund support from Muhimbili University of Health and Allied Sciences under the project named “East Africa Centre of Excellence for Cardiovascular Sciences”. With this letter, I declare that “**The funders had no role in study design, data collection, and analysis, the decision to publish, or preparation of the manuscript**”.
2. **The statement about acknowledging funders that was stated on the acknowledgement section was deleted as you commented**
3. **According to the comments given, I attached a participants biodata in a word document. They also appered in the manuscript as table 1.**

We have no conflict of interests to disclose. As a corresponding author, I confirm that the manuscript has been read and approved for re-submission by all the named authors.

We deeply appreciate your consideration and look forward to receiving any further comments from you and reviewers.

Please adress all correspondence concerning this manuscript to me at [pkasongoro@gmail.com](mailto:pkasongoro@gmail.com)

Sincearly,

Mr. Peter Shirima, MSc

Cardiovascular Nurse,

Muhimbili National Hospital
